# High Hydrostatic Pressure Treatments Improved Properties of Fermentation of Apple Juice Accompanied by Higher Reserved *Lactobacillus plantarum*

**DOI:** 10.3390/foods12030441

**Published:** 2023-01-17

**Authors:** Jing Ma, Yu Wang, Mengya Zhao, Pengyan Tong, Liuqing Lv, Zhenpeng Gao, Jing Liu, Fangyu Long

**Affiliations:** 1 College of Food Science and Engineering, Northwest A&F University, Xianyang 712100, China; 2 Institute of Animal Husbandry and Veterinary Medicine, Guizhou Academy of Agricultural Sciences, Guiyang 550005, China

**Keywords:** apple juice, high hydrostatic pressure, fermentation, phenolic compounds, simulated gastrointestinal condition

## Abstract

This study aimed to assess the feasibility of high hydrostatic pressure (HHP) treatment to obtain high quality juice, and prepared functional apple juice using fermentation technology. The physicochemical properties of HHP (10 min) pasteurized and pasteurized (85 °C, 15 min) apple juices were compared during fermentation. Moreover, the survival of *Lactobacillus plantarum* after fermentation under simulated gastrointestinal conditions was evaluated. Results showed that HHP-treated apple juice had better properties than that of pasteurized in terms of color difference, total phenol content, and antioxidant activity. After fermentation, about 2.00 log CFU/mL increase in viability of cells was observed and there was around 0.8 reduction in pH value, and the antioxidant capacities were also significantly improved. Additionally, the content of caffeic acid, ferulic acid, and chlorogenic acid significantly increased after 24 h of fermentation. The survival of *Lactobacillus plantarum* in simulated gastric fluid reached 97.37% after fermentation. Overall, HHP treatment is expected to be a substitute technology to pasteurization in order to obtain higher quality fermented fruit juice. This study could also be helpful for exploitation of fermented juice.

## 1. Introduction

In recent years, China has jumped to become the world’s largest apple producer with about 50% of the world’s apple planting area and yield. Apples are mass consumption fruit, due to bioactive substances in apples having been shown to reduce the risk of cardiovascular disease [1], cancer [2], obesity [3], and other diseases. To ensure the safety of apple juice, heat treatment is an indispensable treatment. Traditional thermal treatment is still widely used in food processing today. Unfortunately, thermal treatment destroys the heat-sensitive components of the food [4]. Zhao et al. [5] concluded that thermal treatment significantly reduced the content of C_6_ aldehydes and C_6_ alcohols in kiwifruit juice. According to the results of Wibowo et al. [6], pasteurized cloudy apple juice had the formation of off-flavors and the activities of polyphenol oxidase and peroxidase were greatly reduced.

It is necessary to find a novel technology to preserve quality and nutrients during food processing. High hydrostatic pressure (HHP) treatment is a non-thermal technique, and the main advantage over traditional thermal techniques is processing at low temperature in the course of processing, which helps to avoid the loss of biological components [7], such as polyphenols, vitamin C, etc. [8]. Presently, HHP technology is used in food manufacturing to improve storage performance and sensory quality [9]. In view of the high nutritional value of apple juice, HHP treatment can be a potential alternative to traditional thermal processing technologies for the development of safe, tasty, nutritious, and functional food products [4].

In recent years, the production and consumption of fermented juices has received attention. Because of the excellent tolerability in acidic environments, lactic acid bacteria (LAB) have been widely used in the fermentation of fruit and vegetable juices. During the fermentation of probiotics, the sugars, organics, and minerals in apples can be metabolized into other compounds [10], resulting in the unique flavor and efficacy of fermented apple juice [11]. Moreover, LAB have beneficial effect on human health. Among all the LAB, *Lactobacillus plantarum* is the most commonly used strain in fruit and vegetable fermentation [12]. Previous study [13] indicated that *L. plantarum*-fermented juice has the higher radical scavenging activities. Reid et al. [13] showed that consumption of fermented beverages could improve the immunity and increase the number of LAB in the intestine. Han et al. [14] suggested that fermented cloudy apple juice showed potential to balance blood lipid levels in obese mice. Although some studies have reported the effect of LAB fermentation on the quality and functionality of apple juice, there are no studies on the fermentation of HHP-treated apple juice, and few studies have been conducted to test simulated gastrointestinal viability of fermented juices. In this study, HHP treatment and pasteurization were used to treat apple juice and changes in physicochemical properties during fermentation were investigated. Moreover, the survival ability of *L. plantarum* under simulated gastrointestinal conditions was discussed. The aim of the present work is to contrastively analyze the effects of HHP treatment and pasteurization on apple juice and the suitability of fermented apple juice as a functional product.

## 2. Materials and Methods

### 2.1. Materials and Reagent

De Man, Rogosa, and Sharpe (MRS) medium was purchased from Beijing Obosing Biotechnology Co., Ltd. (Beijing, China). *Lactobacillus plantarum* PA01 was isolated from pickle and registered by the China Common Microbial Collections Management Center (CGMCC No.15660). Standards for high-performance liquid chromatography (HPLC) (gallic acid, ferulic acid, catechins, chlorogenic acid, rutin, phloridzin, epicatechin, quercetin, caffeic acid, phloretin) were purchased from Solarbio (Beijing, China). Chromatographic grade acetonitrile (TEDIA, Fairfield, OH, USA) and formic acid (Kemiou, Tianjin, China) were obtained for analysis.

### 2.2. Preparation and Fermentation of Apple Juice

Fresh and ripe “Luo chuan” apples (the starch index is 6.5 to 8) were purchased from a local market, washed, nucleated, and cut into small pieces. Apple pieces were put into a juicer, adding an equal amount (*v*/*w*) of distilled water and crushing, next filtering through four 80 mesh layers. Apple juice was transferred into a sterile polyethylene bag and heat-sealed under a vacuum. Pasteurization (85 °C, 15 min) and high hydrostatic pressure (HHP) treatment were used for apple juice. Pasteurization took place with a water bath (HH–ZK2, Gongyi Yuhua Instrument Co., LTD., Zhengzhou, China) and HHP treatment processing was carried out with different pressures (200, 300, and 400 MPa) at room temperature for 10 min using HHP equipment (HPP.L2–600/10600 MPa, Baotou Kefa High Pressure Technology Co., LTD., Baotou, China).

*L. plantarum* stored at −80 °C was activated three times [15] and used in the fermentation. The juice after pasteurization and the HPP process was inoculated with *L. plantarum*. After inoculation, apple juice was placed into an incubator at 37 °C for 48 h. The initial viable bacterial number of *L. plantarum* in apple juice was about 5.80 log CFU/mL. The results of pretest showed that some of the qualities of apple juice decreased and the quality and nutritional value experienced a peak change after 48 h of fermentation. Therefore, juice samples (100 mL) fermented in glass fermentation cylinders were collected after fermentation for 0, 4, 8, 16, 24, 48 h. Before analysis, the samples were centrifuged at 4 °C and 6000× *g* for 10 min.

### 2.3. Total Acid and pH Values

We used the acid–base titration method to determine the total acid, and expressed it as the malic acid conversion coefficient (0.067). A pH meter (Five Easy Plus, Shenzhen Weifeng Instrument Co., Ltd., Shenzhen, China) was used to determine the pH values of fermented apple juice [15]. All measurements were taken three times.

### 2.4. Measurement of Color

The color of fermented apple juice was measured using a colorimeter (CS–820, Hangzhou Color Spectrum Technology Co., Hangzhou, China). The color parameters (L*, a* and b*) were tested by setting the light source of D65. The color difference (ΔE) was calculated by the following formulas:

$$\Delta E=\sqrt{{\left(L^{*}-L_{0}^{*}\right)}^{2}+{\left(a^{*}-a_{0}^{*}\right)}^{2}+{\left(b^{*}-b_{0}^{*}\right)}^{2}}$$

where L_0_*, a_0_*, and b_0_* represent the color values of unpasteurized apple juice, and the color values of fermented apple juice were indicated by L*, a*, and b*, respectively.

### 2.5. Determination of Viable LAB Cells

The viable cell numbers were determined by the plate counting method. Specifically, 1 mL of fermented juice was diluted serially with sterile saline to 10^4^–10^6^ dilutions, Inoculating 0.2 mL of diluted sample on MRS agar, and incubating at 37 °C for 36–48 h. After incubation, characteristic colonies were counted [15]. The viability of LAB was expressed as log CFU/mL.

### 2.6. Total Phenol Content and Polyphenolic Compounds

The total phenol content of fermented apple juice was conducted as previously described by Yang et al. [16] with some modifications. Specifically, 1.0 mL of apple juice (20-fold dilution) was mixed with 1.0 mL of 7.5% (*w*/*v*) Na_3_CO_2_. Then, 3.0 mL of Folin–Ciocâlteu (0.5 mol/L) reagent was added. The mixture was left for 60 min in 30 °C water bath in the dark and the absorbance measured at 765 nm.

After a comprehensive evaluation of apple juice from different treatments, we monitored the changes of phenolic monomers in apple juice from 200 MPa treatment. Prior to the assay, phenols in fermented apple juice were extracted by ethyl acetate. An equal amount of ethyl acetate was added to 12 mL of apple juice and the upper organic phase was collected after sufficient extraction. The extraction was repeated three times. The organic phases were merged for rotatory evaporation at 45 °C under vacuum and re-dissolved with methanol (4 mL). Before injection, the sample was filtered by 0.22 μm organic phase filter membranes.

Polyphenolic compounds were analyzed using high performance liquid chromatography (HPLC, Shimadzu, Japan). The main chemical components were separated on a C_18_ chromatographic column (ZORBAX SB–C18, 250 mm × 4.6 mm, 5 μm, Agilent Technologies Inc., Santa Clara, CA, USA) at flow rate 1.0 mL/min and detection with the wavelength 280 nm. The mobile phase consisted of 0.1% formic acid in water (solvent A) and acetonitrile (solvent B). The gradient elution procedure of phase B was: 0–5 min, 5%; 5–25 min, 12%; 25–40 min, 30%; 40–50 min, 45%; 50–60 min, 5%. The oven temperature was 32 °C.

### 2.7. Antioxidant Activities

#### 2.7.1. ABTS-Based Scavenging Activity

ABTS radical scavenging activity was measured following Zhu et al. [17] with some modifications. Specifically, the ABTS radical cation was prepared by mixing 7.4 mM of ABTS stock solution with potassium persulphate (4.6 mM) in a 1:1 ratio. The mixture was stored in the dark for 12–16 h. Before use, the ABTS solution was diluted with distilled water to an absorbance of 0.70 ± 0.02 at 734 nm. Adding 200 μL of sample solution into diluted ABTS solution (800 µL), the absorbance was measured after 6 min with Spark microplate reader (Tecan Austria GmbH, Grödig, Austria). ABTS radical scavenging activity was calculated as follows:

$$\mathrm{Scavenging}\;\mathrm{rate}\;\left(\%\right)=\frac{\left(A_{2}-A_{1}\right)}{A_{2}}\times 100$$

where A_2_ is absorbance of the control group and A_1_ is absorbance of the sample group.

#### 2.7.2. DPPH Scavenging Ability

According to method of Karioti at al. [18] with a slight modification, DPPH scavenging ability was determined. In particular, 500 μL of DPPH–ethanol solution (0.1 mM) was mixed with 500 μL of sample solution, followed by reacting in the dark for 30 min. The absorbance was measured at 517 nm. Each sample was tested thrice in parallel. The percentage of DPPH scavenging ability was calculated as follows:

$$\mathrm{Scavenging}\;\mathrm{rate}\;\left(\%\right)=\left(1-\frac{A_{1-}A_{2}}{A_{0}}\right)\times 100$$

where A_0_ is the background absorbance of DPPH solution, A_1_ is absorbance of the sample experimental group, and A_2_ is the background absorbance of sample.

### 2.8. Survival of L. plantarum Fermentation in Apple Juice under Simulated Gastrointestinal Conditions

After a comprehensive evaluation of apple juice from different treatments, we determined the viability of *L*. *plantarum* in fermented apple juice treated with 200 MPa.

Simulated gastric fluid (SGF) and simulated intestinal fluid (SIF) solutions were prepared following the method of Roberts et al. [19]. The SGF was prepared by adding 2 g of NaCl in 7 mL of HCl (12 M), then the volume was up to 1 L with distilled water, and adjusted the pH to 2.5 with NaOH (1 M). Next, 1 mL of fermented apple juice and *L. plantarum* cell solution (6.0 log CFU/mL) was suspended in 9.0 mL of sterilized and pre-warmed SGF, then incubated in 37 °C water bath for 60 min. Samples were collected at 0, 10, 20, 30, 40, 50, and 60 min intervals and the viable cells were counted [20].

The SIF was made by dissolving 6.8 g of KH_2_PO_4_ in 250 mL of distilled water, and 77 mL of NaOH (0.2 M) was added. Volume was fixed to 1 L with distilled water. The fermented apple juice (1 mL) and *L. plantarum* cell solution (6.0 log CFU/mL) was suspended in 9.0 mL of sterilized and pre-warmed SIF, then incubated in water bath at 37 °C for 120 min. Samples were collected at 30-min intervals until 120 min of incubation, then the viable cells were counted [20].

### 2.9. Statistical Analysis

One-way analysis of variance (ANOVA) was performed on the triplicate experiments using SPSS 20.0 software, *p* < 0.05 was considered statistically significant. Pearson’s correlation analysis for variables with normal distribution was used in correlation analysis.

## 3. Results and Discussion

### 3.1. Comparing the Effect of Treatments on Quality Properties of Apple Juice

**Figure 1:**
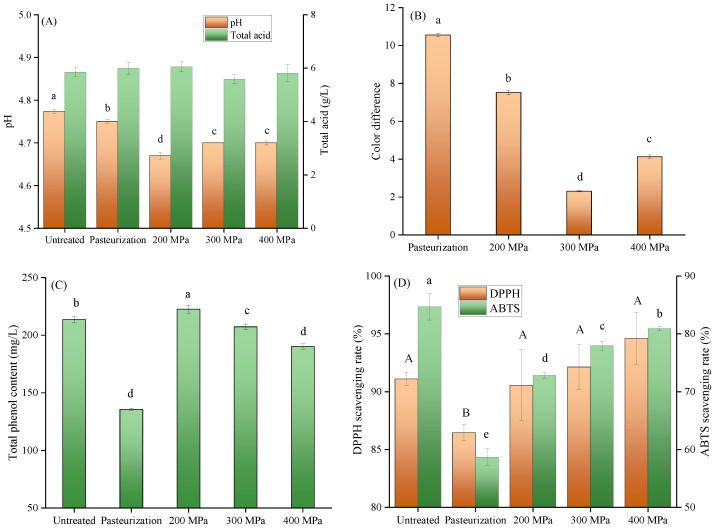
Effects of different treatments on (**A**) total acid and pH values, (**B**) color difference, (**C**) total phenol content, and (**D**) DPPH and ABTS scavenging rate of apple juice. HHP treatment at 200 and 400 MPa for 10 min; pasteurization (85 °C, 15 min). Different small letters indicate significant differences (*p* < 0.05) between different samples, and different capital letters indicate significant differences (*p* < 0.05) in DPPH. Experiments were performed in triplicate; vertical bars indicate ± SD (*p* < 0.05).

The pH and total acid of untreated apple juice were 4.71 and 5.85 g/L, respectively (Figure 1A). All treatments significantly (*p* < 0.05) reduced the pH of apple juice compared to untreated apple juice. It can be seen from Figure 1B that all apple juices produced visual color changes after processing. In contrast, the effect of HHP treatment on color was milder than that of pasteurization (*p* < 0.05), which may probably be because heat treatment denatured the proteins in the juice to produce insoluble denatured proteins, resulting in color change [6]. It has been proven that HHP treatment had minimal effect on covalent bonds of compounds with low molar mass, color can be preserved [6]. By comparing apple juices treated with different pressures, it was found that the color difference decreased with increasing pressure, but when the pressure was increased to 400 MPa, the color difference increased. This may be because the high pressure affects the covalent bonds of the color compounds. Comparing with pasteurization, HHP treatment can preserve as much of the polyphenol in apple juice as possible (Figure 1C). Heat treatment accelerated binding to proteins and led to degradation of phenolic compounds [21], while HHP treatment affected the structure of cells and allowed more material to escape [22]. There was no significant difference in the DPPH radical scavenging rate of apple juice with different HHP treatments (*p* > 0.05), similar to the results of Chaikham et al. [23], who found no significant effect of HHP treatment (100–600 MPa) on DPPH radical scavenging rate of lychee syrup. As can be seen from Figure 1D, HHP treatment can effectively maintain the DPPH and ABTS radical scavenging ability of apple juice. To sum up, in order to retain the nutritional value and biological activity of apple juice, 200 MPa and 400 MPa HHP treatments were selected to carry out subsequent experiments.

### 3.2. Effect of Fermentation on Physicochemical Properties of Apple Juice with Different Treatments

**Figure 2:**
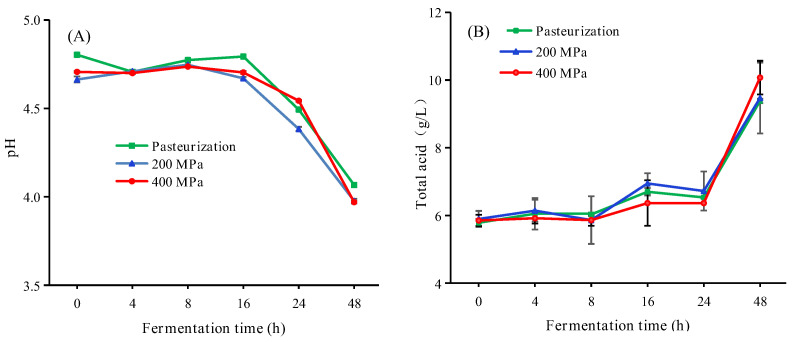
Changes in pH value (**A**) and total acid (**B**) of apple juice with different treatments during fermentation. HHP treatments (200 or 400 MPa, 10 min); pasteurization (85 °C, 15 min). Experiments were performed in triplicate; vertical bars indicate ± SD (*p* < 0.05).

During the fermentation, the pH value of apple juice showed a downward trend and the total acid was on the rise as a whole (Figure 2). There were no significant differences among all treatments, which was consistent with the previous reports [24]. The pH values of apple juice, treated with pasteurization and HHP treatment, were about 4.70 and decreased significantly (*p* < 0.05) after 24 h of fermentation. As reported by Yang et al. [15], fermentation by LAB usually leads to a decrease in pH value of juice. After 48 h of fermentation, the total acid content of apple juice showed an overall upward trend (Figure 2B), and there was no significant difference between pasteurized and HHP treated cloudy apple juice [25]. LAB uses malic acid for growth and metabolism, converting it to pyruvic acid and then to lactic acid, which enriches the flavor and taste of apple juice. Furthermore, LAB consumes carbohydrates to produce numerous organic acids, which reduces the pH value of juice, effectively prevents bacterial contamination, and improves the biological stability of beverages [26]. Thus, it has high application value for improving the shelf life of apple juice by fermentation with LAB.

### 3.3. Changes in Color

The effect of fermentation on color characteristics of apple juice was investigated by color features (L*, a*, and b*) and the color difference (ΔE). The results (Table 1) revealed increases in L* and ΔE, a decrease in a^*^ and a small scale of variation in b* during the fermentation. Compared with the pasteurization, ΔE of HHP treated apple juices were lower when fermentation for the same time. Overall, the color properties of fermented apple juice with 200 MPa treatment was relative to the best, followed by the 400 MPa treatment. In contrast, the color properties of HHP treated apple juice was superior to pasteurized. The proteins in the juice after heat treatment were denatured during the process to produce insoluble denatured proteins, so the L* value of pasteurized apple juice is lower. At the same time, higher temperature can cause certain substances to change or react.

### 3.4. Variations of Viable Cell Number during Fermentation

**Figure 3:**
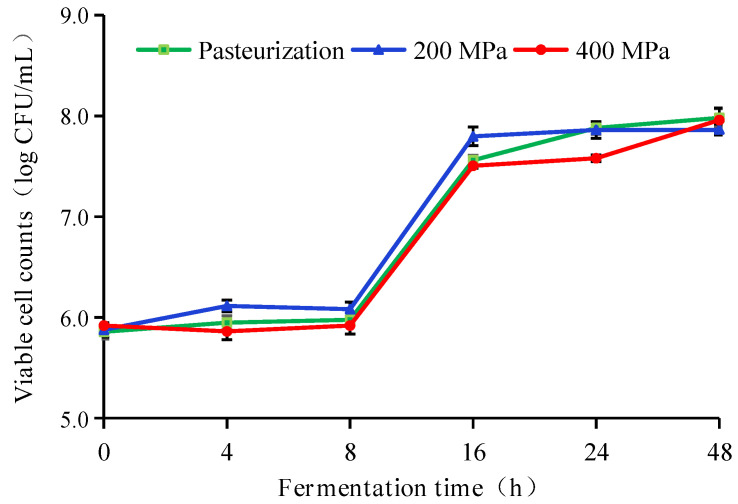
Variations of viable cell number of different treated apple juices during fermentation. HHP treatments (200 or 400 MPa, 10 min); pasteurization (85 °C, 15 min). Experiments were performed in triplicate; vertical bars indicate ± SD (*p* < 0.05).

There were similar trends in viable cell numbers of pasteurized and HHP pasteurized apple juices during fermentation. After fermentation for 8 h, the number of viable cells in apple juice increased rapidly and then stabilized after 16 h, which has a similar trend with the results of Han et al. [27]. As can be seen from Figure 3, the viable cell number of *L. plantarum* was above 7.5 log CFU/mL after fermentation for 16–48 h, which not only could prove that apple juice could be used as a suitable substrate for fermentation of *L. plantarum*, but also met the requirement for the viable cell number.

### 3.5. Comparative Analyses of Antioxidant Activity of Different Treated Apple Juices during Fermentation

**Figure 4:**
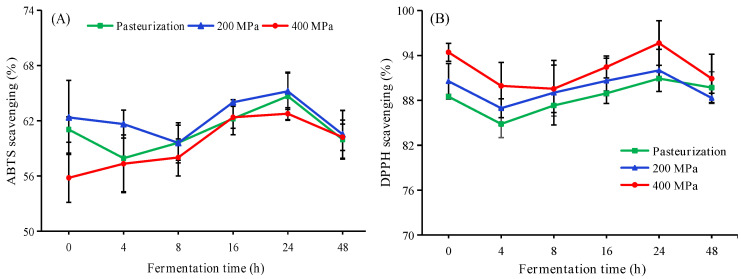
ABTS scavenging ability (**A**) and DPPH scavenging ability (**B**) of different treated apple juice during fermentation. HHP treatments (200 or 400 MPa, 10 min); Pasteurization (85 °C, 15 min). Experiments were performed in triplicate; vertical bars indicate ± SD (*p* < 0.05).

**Figure 5:**
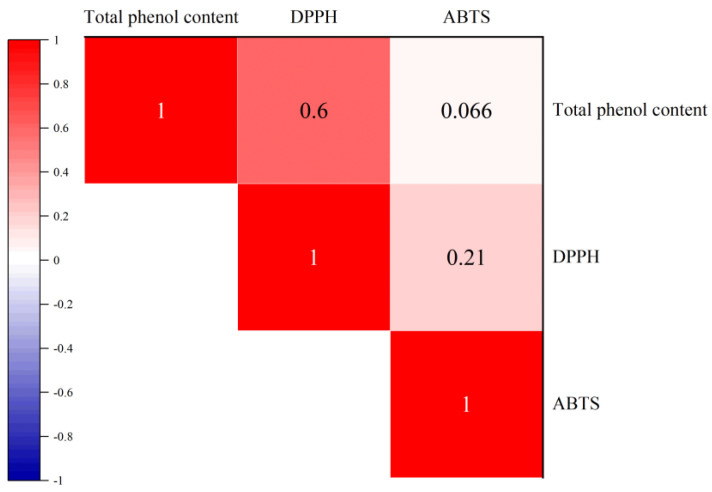
Correlation coefficients (*r*) among total phenol content, DPPH scavenging ability, and ABTS scavenging ability.

The polyphenols and flavonoids in apple juice have strong antioxidant activity. The effect of fermentation on antioxidant activity of apple juice are shown in Figure 4. Results suggested that the ABTS scavenging ability of 200 MPa HHP-treated apple juice was higher than others during the process of fermentation, and the ABTS scavenging increased continually to 65.20 ± 2.58% after 24 h (Figure 4A). Zhu et al. [25] reported that fermentation can enhance the ABTS scavenging activity of black apples. DPPH radical scavenging ability of HHP treated apple juice during fermentation can be seen from Figure 4B. The scavenging rate of DPPH radicals decreased sharply during the initial 4 h of fermentation but increased continuously by 24 h. This is probably because fermentation by *L. plantarum* can promote the utilization of polyphenol compounds with proton donor characteristics [25]. Zhou et al. [28] reported that *L. plantarum* improved the antioxidant activity of kiwifruit pulp by fermentation at 37 °C for 28 h. Pearson coefficient (Figure 5) indicated that DPPH (*r* = 0.600, *p* < 0.05) and ABTS (*r* = 0.066, *p* > 0.05) scavenging ability was positively correlated with total phenol content during the fermentation, which may indicate that phenol content was correlated with the antioxidant ability of apple juice [16,25].

It was proposed by Tkacz et al. [29] that *L. plantarum* affects the transformation or protection of bioactive compounds, which actively participates in the enhancement of antioxidant activity. Previous studies have mostly confirmed the active role of some *L. plantarum* in antioxidant activity, including on mulberry [13], kiwifruit [28], sea buckthorn juice [29], and mango juice [30].

### 3.6. Total Phenol Content and Polyphenolic Profiles

**Figure 6:**
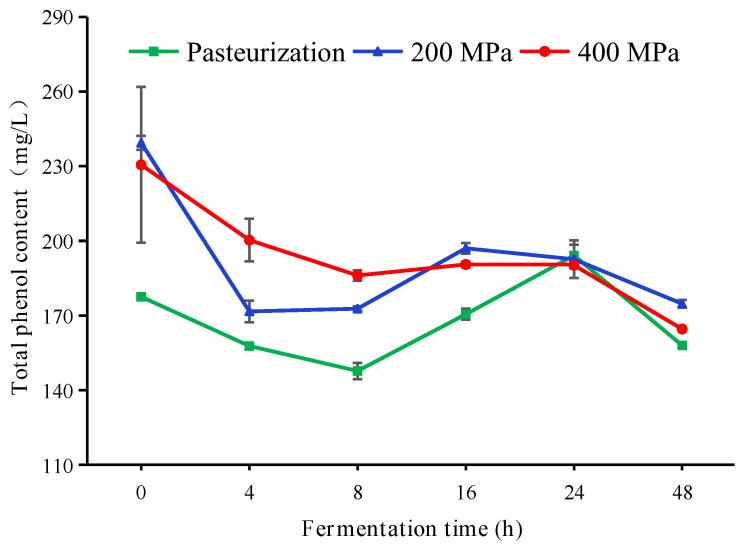
Total phenol content of apple juice with different treatments during fermentation. HHP treatments (200 or 400 MPa, 10 min); pasteurization (85 °C, 15 min). Experiments were performed in triplicate; vertical bars indicate ± SD (*p* < 0.05).

The changes in the total phenol content of apple juice during fermentation were shown in Figure 6. The phenol content of HHP-treated apple juice was significantly higher than that of pasteurized in the pre-mid fermentation period (*p* < 0.05). During the pre-fermentation period, the phenol content showed a decreasing trend, which may be due to the binding of proteins in apple juice with phenol could produce insoluble complexes [31]. The phenol content decreased significantly (*p* < 0.05) after 24 h of fermentation. Certain concentration of phenol had an antibacterial effect, thus LAB decomposed phenol compounds in order to sustain its growth [32]. The total phenol content in pasteurized apple juice was lower during the fermentation process, probably because the high temperature promoted the decomposition and conversion of polyphenols [21], and there was no significant difference in the total phenol content between 200 MP and 400 MP treated apple juice at the later stage of fermentation (*p* > 0.05).

Higher phenol content leads to the hypothesis that fermented apple juice had greater potential hyperglycemic and hypolipidemic activities [11]. A comprehensive evaluation of different treated apple juices revealed that 200 MPa processed apple juice had higher total phenol content and antioxidant activity. Therefore, variations in phenolic monomers of 200 MPa treated apple juice were monitored.

**Figure 7:**
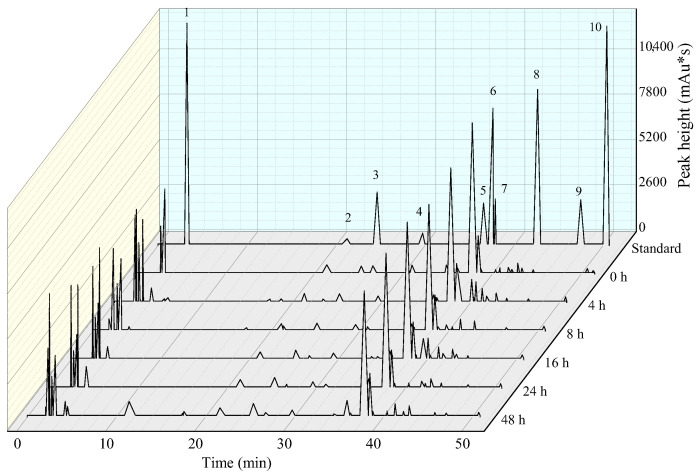
Chromatograms of polyphenolic profiles in 200 MPa treated apple juice with different fermentation time. 1—Gallic acid; 2—catechins; 3—caffeic acid; 4—epicatechin; 5—chlorogenic acid; 6—ferulic acid; 7—rutin; 8—phlorizin; 9—quercetin; 10—phloretin.

The polyphenols In fruits have many positive effects on human health, such as quercitrin, phloridzin, chlorogenic acid, epicatechin, and catechin [33]. The fermentation process caused variations in the phenolic acid and flavonoid profiles in apple juice. The content of gallic acid, catechins, caffeic acid, epicatechin, chlorogenic acid, ferulic acid, rutin, phlorizin, quercetin, and phloretin are summarized in Figure 7 and Table 2. We found an increase in caffeic acid and chlorogenic acid during fermentation. Chlorogenic acid has a hypocholesterolemic effect [34]. Zhang et al. [35] demonstrated that caffeic acid contributes to ABTS free radical scavenging activity. Ferulic acid is a precursor substance for the synthesis of aroma components by microorganisms. The production of aroma components during fermentation may led to a decrease in ferulic acid content. Rutin, the main flavonoid polyphenol in apple juice, had no significant change during the 24 h of fermentation (*p* > 0.05). The rutin content in apple juice before fermentation was 3.77 mg/L, while the results of Li et al. [36] indicated that before vaccination, the rutin content in apple juice was 0.50 mg/L. This may be due to the high content of rutin in the peel, whereas the apples used in this study were unpeeled. Quercetin can relieve cough, resolve phlegm, and lower blood pressure [37]. Quercetin and rhizothein had maximum values at 16 h of fermentation. Li et al. [38] pointed out that the content of quercetin in apple juice significantly increased during the fermentation. Meanwhile, quercetin and phloretin have good oxidation resistance, which can increase the activities of CAT, GSH–Px, and SOD in rats, thus improving oxidative stress [39].

Hydrolytic enzymes in *Lactobacillus* hydrolyze complex polyphenol compounds into simple forms and release bound polyphenol compounds from plant cell walls [40]. The proper fermentation time is a practical and effective method to improve the function of apple juice. At 24 h of fermentation, the effect of fermentation on the forward synthesis of polyphenol monomers was greater than the effect of reverse depletion.

### 3.7. Viability of L. plantarum after Fermentation in Apple Juice under Simulated Gastrointestinal Conditions

**Figure 8:**
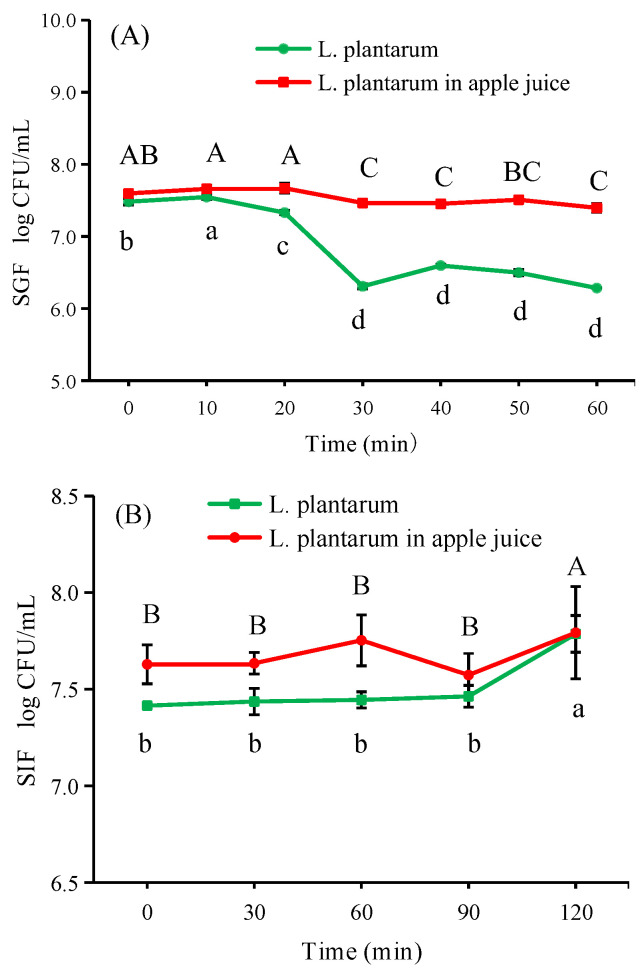
Variations of viability of *L. plantarum* suspension and *L. plantarum* in fermented apple juice under simulated gastric fluid (**A**) and simulated intestinal fluid (**B**) conditions. Experiments were performed in triplicate; vertical bars indicate ± SD (*p* < 0.05). Different small letters and capital letters indicate significant differences (*p* < 0.05) of *L. plantarum* suspension and *L. plantarum* in fermented apple juice, respectively.

Based on the above results, 200 MPa treated apple juice fermented for 24 h was applied for viability tests in simulated gastrointestinal fluid. Coghetto et al. [41] and Xu et al. [42] found that the alkaline environment in simulated intestinal fluid (SIF) could cause cell death for some probiotic strains; however, in our study, it had minimal adverse effect on *L. plantarum* strains (Figure 8B). The viable cell number after SIG treatment increased by 0.16 and 0.22 log CFU/mL for *L. plantarum* after fermentation and free *L. plantarum* cells, respectively. The time for liquid food to empty from the stomach was about 20 min in accordance with Chen et al. [20]. Accordingly, fermented apple juice with *L. plantarum* was incubated in simulated gastric fluid (SGF) for 60 min to determine its viability. During the viability assay in SGF, the number of free *L. plantarum* cells reduced significantly when exposed for 20 min (Figure 8A). The viable cell number of *L. plantarum* was reduced by 1.20 log CFU/mL after 60 min of incubation, while the viability of *L. plantarum* after fermentation decreased from 7.60 to 7.40 log CFU/mL, the survival reached 97.37%. Fermentation of *L. plantarum* in apple juice enhanced its viability when exposed to SGF conditions. This may be due to the protection offered by the bacteria in combination with the components in apple juice. *L. plantarum* can adsorb the anthocyanin glucosides and quercetin glycosides [43]. Roberts et al. [19] suggested that unfiltered apple juice containing natural pectin may have the greatest effect on preserved cell viability under SGF and SIF conditions. In this study, unpeeled apple juice was prepared, gauze filtration may filter out most of the pectin, but there may still be some pectin present in the filtered juice. Adsorption of substances by *L. plantarum* is like an encapsulation of the bacteria, providing a protective effect. Chen et al. [42] found that high survival of microencapsulated *Lactobacillus bulgaricus* under simulated gastrointestinal conditions.

## 4. Conclusions

HHP treatment is more suitable than pasteurization as a pretreatment technology for fermented apple juice, and 200 MPa treatment is better than 400 MPa. The results showed that the fermented apple juice produced by HHP process had low color difference, high antioxidant activity, and high nutritional value. After 24 h of fermentation, the color difference, the number of *L. plantarum*, and antioxidant activity of 200 MPa-produced apple juice increased. The content of caffeic acid, ferulic acid, and chlorogenic acid also significantly increased. In addition, fermentation enhanced the viability of *L. plantarum* when exposed to SGF. Therefore, high pressure treatment combined with *L. plantarum* fermentation is the better processing method of apple juice in terms of quality and nutritional composition.

## Figures and Tables

**Table 1:** Changes in color properties of different treated apple juices as influenced by fermentation.

Treatment	Fermentation Time (h)	L*	a*	b*	∆E
Pasteurization	0	46.31 ± 0.02 ^fC^	20.89 ± 0.03 ^aC^	65.66 ± 0.06 ^cC^	7.33 ± 0.03 ^fA^
	4	47.28 ± 0.04 ^dB^	20.37 ± 0.04 ^bC^	63.74 ± 0.06 ^dC^	7.54 ± 0.08 ^eA^
	8	46.81 ± 0.03 ^eC^	20.38 ± 0.02 ^bC^	66.19 ± 0.18 ^bC^	9.07 ± 0.09 ^dA^
	16	51.74 ± 0.03 ^cB^	18.10 ± 0.04 ^cC^	67.39 ± 0.15 ^aC^	10.98 ± 0.07 ^cA^
	24	53.19 ± 0.01 ^bA^	16.74 ± 0.03 ^dC^	67.50 ± 0.13 ^aC^	12.87 ± 0.01 ^bA^
	48	55.04 ± 0.03 ^aC^	15.19 ± 0.02 ^eC^	65.52 ± 0.02 ^cC^	15.62 ± 0.03 ^aA^
200 MPa	0	48.01 ± 0.02 ^fA^	25.31 ± 0.05 ^aA^	69.71 ± 0.18 ^eB^	2.88 ± 0.05 ^fC^
	4	47.91 ± 0.02 ^eA^	24.54 ± 0.00 ^bA^	70.53 ± 0.09 ^dA^	3.35 ± 0.02 ^eC^
	8	48.94 ± 0.01 ^dA^	23.91 ± 0.02 ^cA^	71.24 ± 0.01 ^cA^	4.67 ± 0.06 ^dB^
	16	52.56 ± 0.02 ^cA^	20.78 ± 0.03 ^dB^	72.15 ± 0.08 ^aA^	9.53 ± 0.05 ^cB^
	24	52.67 ± 0.04 ^bB^	19.59 ± 0.00 ^eB^	71.46 ± 0.16 ^bcA^	10.27 ± 0.07 ^bB^
	48	56.44 ± 0.01 ^aB^	16.01 ± 0.03 ^fB^	71.64 ± 0.10 ^bA^	15.44 ± 0.04 ^aB^
400 MPa	0	47.86 ± 0.01 ^fB^	24.85 ± 0.03 ^aB^	70.17 ± 0.14 ^bA^	3.05 ± 0.05 ^fB^
	4	47.95 ± 0.01 ^eA^	24.03 ± 0.02 ^bB^	69.50 ± 0.15 ^cB^	3.70 ± 0.03 ^eB^
	8	48.39 ± 0.00 ^dB^	23.44 ± 0.01 ^cB^	70.46 ± 0.11 ^abB^	4.46 ± 0.03 ^dC^
	16	50.38 ± 0.02 ^cC^	22.14 ± 0.02 ^dA^	69.62 ± 0.17 ^cB^	6.73 ± 0.04 ^cC^
	24	50.61 ± 0.01 ^bC^	21.52 ± 0.02 ^eA^	70.19 ± 0.17 ^bB^	7.34 ± 0.02 ^bC^
	48	56.71 ± 0.02 ^aA^	17.09 ± 0.03 ^fA^	70.66 ± 0.13 ^aB^	14.82 ± 0.02 ^aC^

Values are means and standard errors of three determinations. Different letters (a–f) indicate significant differences (*p* < 0.05) among different fermentation time for apple juice, and (A–C) suggest significant differences (*p* < 0.05) between different method with same fermentation time.

**Table 2:** Variations in polyphenolic profiles of 200 MPa treated apple juice during fermentation.

Category/ Fermentation Time	Content (mg/L)					
	0 h	4 h	8 h	16 h	24 h	48 h
Gallic acid	4.30 ± 0.00 ^c^	4.12 ± 0.08 ^d^	4.52 ± 0.05 ^b^	3.94 ± 0.00 ^e^	3.64 ± 0.05 ^f^	4.89 ± 0.00 ^a^
Catechins	4.26 ± 0.00 ^a^	3.68 ± 0.02 ^ab^	3.25 ± 0.04 ^b^	3.49 ± 0.02 ^b^	3.90 ± 0.16 ^ab^	3.78 ± 0.45 ^ab^
Caffeic acid	1.44 ± 0.00 ^d^	1.49 ± 0.01 ^cd^	1.47 ± 0.00 ^cd^	1.52 ± 0.01 ^c^	1.62 ± 0.01 ^b^	1.70 ± 0.04 ^a^
Epicatechin	2.83 ± 0.00 ^a^	2.34 ± 0.02 ^c^	2.32 ± 0.06 ^d^	2.45 ± 0.01 ^b^	2.36 ± 0.06 ^bc^	2.44 ± 0.02 ^bc^
Chlorogenic acid	0.24 ± 0.00 ^d^	0.27 ± 0.03 ^d^	0.30 ± 0.02 ^d^	0.55 ± 0.03 ^c^	0.75 ± 0.10 ^b^	1.42 ± 0.28 ^a^
Ferulic acid	4.47 ± 0.00 ^a^	4.01 ± 0.11 ^c^	3.98 ± 0.05 ^c^	4.35 ± 0.01 ^ab^	4.17 ± 0.01 ^bc^	3.91 ± 0.14 ^c^
Rutin	3.77 ± 0.00 ^b^	3.76 ± 0.12 ^b^	3.68 ± 0.00 ^b^	3.76 ± 0.03 ^b^	3.71 ± 0.01 ^b^	4.08 ± 0.06 ^a^
Phlorizin	1.12 ± 0.00 ^ab^	1.11 ± 0.01 ^b^	1.13 ± 0.00 ^ab^	1.15 ± 0.00 ^a^	1.14 ± 0.01 ^ab^	1.14 ± 0.02 ^ab^
Quercetin	-	1.58 ± 0.00 ^ab^	1.58 ± 0.00 ^b^	1.59 ± 0.00 ^a^	1.57 ± 0.00 ^b^	1.57 ± 0.01 ^b^
Phloretin	1.04 ± 0.00 ^c^	1.05 ± 0.00 ^ab^	1.05 ± 0.00 ^ab^	1.06 ± 0.00 ^a^	1.05 ± 0.00 ^ab^	1.05 ± 0.00 ^b^

Values are means and standard errors of three determinations. Values with the different letters within one line are significantly different (*p* < 0.05).

## Data Availability

The data presented in this study are available on request from the corresponding author.
